# Prosthetic Rehabilitation in Children: An Alternative Clinical Technique

**DOI:** 10.1155/2013/512951

**Published:** 2013-09-24

**Authors:** Nádia Carolina Teixeira Marques, Carla Vecchione Gurgel, Ana Paula Fernandes, Marta Cunha Lima, Maria Aparecida Andrade Moreira Machado, Simone Soares, Thais Marchini Oliveira

**Affiliations:** ^1^Department of Pediatric Dentistry, Orthodontics and Public Health, Bauru School of Dentistry, University of São Paulo, Al. Octávio Pinheiro Brisolla 9-75, 17012-901, Bauru-SP 73, Brazil; ^2^Matrix Dynamics Group, Faculty of Dentistry, University of Toronto, Toronto, ON, Canada; ^3^Department of Prosthetic Dentistry, Bauru School of Dentistry, University of São Paulo, São Paulo, Bauru, Brazil; ^4^Hospital for the Rehabilitation of Craniofacial Anomalies, University of São Paulo, São Paulo, SP, Brazil

## Abstract

Complete and partial removable dentures have been used successfully in numerous patients with oligodontia and/or anodontia. However, there is little information in the literature regarding the principles and guidelines to prosthetic rehabilitation for growing children. This case report describes the management of a young child with oligodontia as well as the treatment planning and the prosthetic rehabilitation technique.

## 1. Introduction

Prosthetic treatment can play an important role when treating children whose dentition fails to develop normally. The congenital absence of teeth is one of the most frequent reasons for the need of complete and partial dentures for young children [1]. Some genetic conditions, such as hypohidrotic ectodermal dysplasia and Papillon-Lefevre syndrome, may cause oligodontia or anodontia. Premature loss of primary teeth due to grossly caries may be another reason for the need of complete dentures for preschool children [2].

Treatment of patients with oligodontia can challenge the clinician not only because patients present a great number of congenitally missing teeth, or even anodontia, but also because of the age they are usually referred for rehabilitation [3]. Several treatment strategies have been reported for the management of patients suffering from anodontia, oligodontia, and malformed teeth. Conventional removable complete or partial dentures are usually the most frequent prosthetic treatment plan for these young patients [3–6].

Since alveolar bone development is dependent on the presence of teeth, children with oligodontia or anodontia have atrophy of the alveolar bone and consequently little or no bone ridge to support dentures [5, 7]. Indeed being edentulous has many consequences, speech impairment, deforming lingual habits, and a poor nutrition, due to the fact that mastication is difficult or impossible [8, 9]. Furthermore, primary teeth are necessary for the acquisition and maturation of diverse functions, which are important for normal growth [10]. Also, in these patients the poor appearance of the teeth can affect self-esteem, which challenges the clinician [5, 8]. 

Complete and partial removable dentures have been used successfully in numerous patients with oligodontia and/or anodontia. However, there is little information in the literature regarding the principles and guidelines to prosthetic rehabilitation for growing children. The rehabilitation of a child's dentition with a removable prosthesis is more complex and time consuming than in adults. All these factors should be considered during fabrication in order to provide a prosthesis that is functional, esthetic, and age appropriate [11]. This case report describes the treatment planning of a young child with oligodontia as well as the prosthetic rehabilitation technique.

## 2. Case Report

A 5-year-old boy presented to the Department of Pediatric Dentistry of our university due to the lack of teeth and problems with speech and mastication. The mother related that an evaluation by a pediatrician resulted in the diagnosis of hypohidrotic ectodermal dysplasia (HED).

The extraoral examination revealed the typical facial physiognomy of HED with prominent forehead and ears, protuberant and everted lips, and a sunken nasal bridge (“saddle nose”). The patient also presented with sparse scalp hair, missing eyelashes and eyebrows, and severe hypohidrosis. The skin appeared dry with hyperpigmentation around the eyes and the mouth. A diminished lower facial height contributed to a senile facial expression.

**Figure 1:**
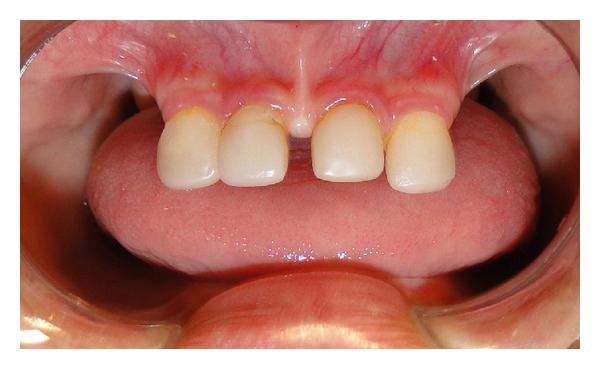
Initial intraoral view showing the maxillary incisors.

**Figure 2:**
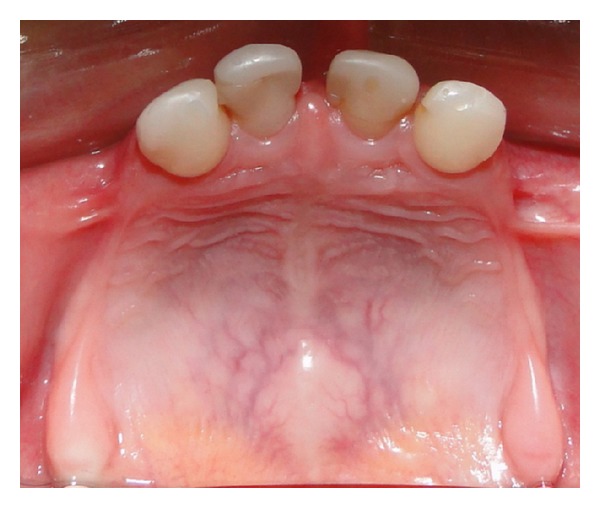
Occlusal view of the maxilla.

**Figure 3:**
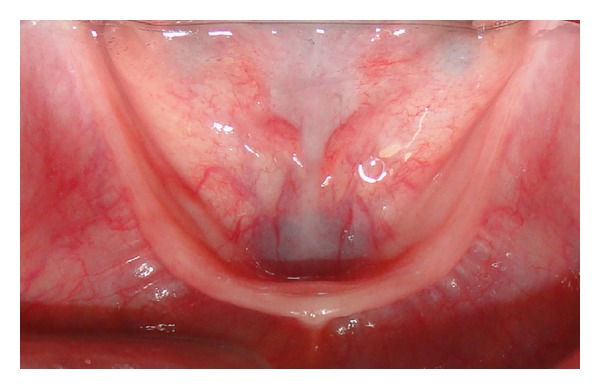
Occlusal view of the mandible.

**Figure 4:**
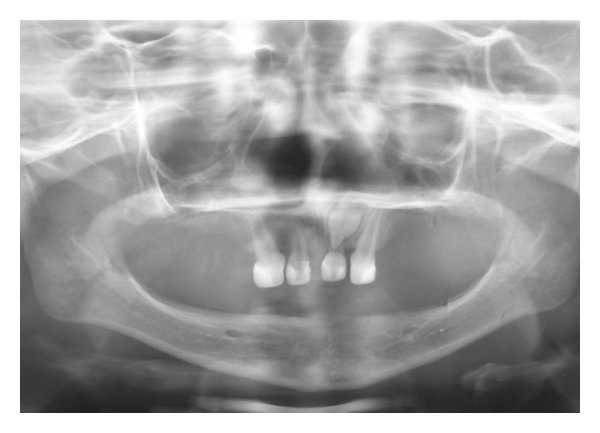
Panoramic radiograph showing oligodontia and lack of alveolar bone development.

Clinical and radiographical examination revealed a partially edentulous maxilla and a completely edentulous mandible. In the maxilla only the primary maxillary central and lateral incisors were present. The primary maxillary central and lateral incisors had apparently been previously restored using resin strip crowns, because these teeth are often conical in shape in ectodermal dysplasia (Figures 1, 2, and 3). Both alveolar ridges were poorly developed with normal appearance of alveolar mucosa. The palate was shallow and the oral mucosa was healthy with a slight dry appearance. The tongue was relatively large, with no signs of macroglossia. Radiographic examination revealed the complete absence of permanent tooth germs, except the permanent maxillary left central incisor (Figure 4).

In order to improve the appearance, mastication, and speech, the treatment plan included a removable partial upper denture and a complete lower denture. The parents were informed about the procedures involved in the fabrication of the dental prostheses and the need for continuing reevaluation and remaking of the oral prostheses as the alveolar ridges and oral environment changed due to growth.

**Figure 5:**
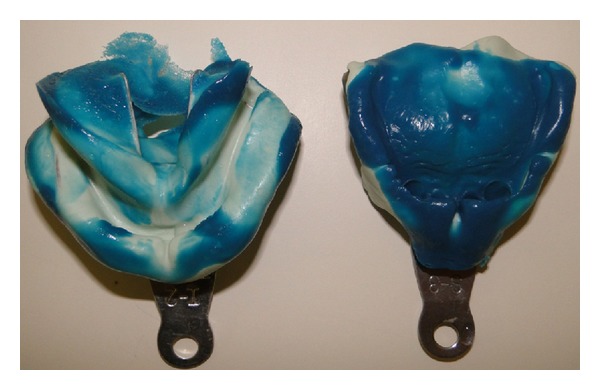
Silicone maxillary and mandibular impressions.

Behavioral management techniques, such as tell-show-do, were used throughout the procedures for construction of the dentures. The smallest stock tray was selected, due to limited mouth opening and developing swallowing mechanisms. First the mandibular impression was made and then the maxillary impression in order to decrease anxiety in the patient. Impressions were made in two stages but in the same session. We used trays and modeling with impression compound silicone putty (Express XT, 3M ESPE, são Paulo, SP, Brazil). The second modeling was made with silicone soft (Figure 5). The first impression was necessary to ensure that vestibular sulcus was properly printed with correct height and width. 

During hardening of the material, the lips and cheeks were manipulated to shape the buccal and labial aspects of the impression. The child was asked to protrude the tongue to raise the floor of the mouth. The final casts were made in dental stone.

**Figure 6:**
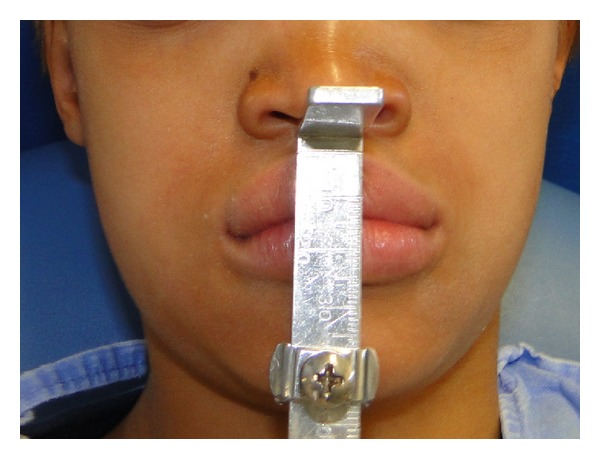
Initial frontal view of the patient. Note the diminished OVD.

**Figure 7:**
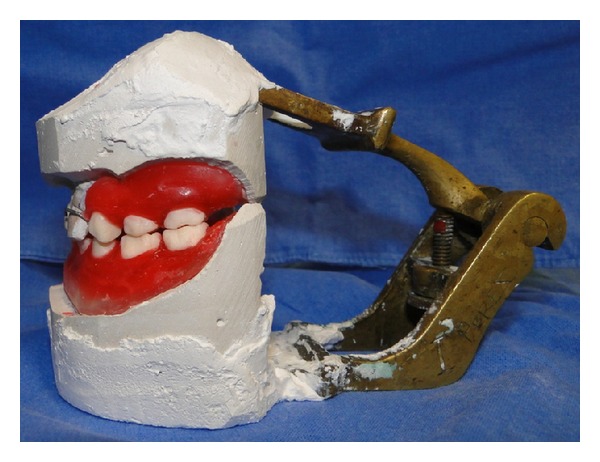
Working casts mounted in a simple articulator.

Shellac base plates were adapted on upper and lower casts, and wax occlusal rims were then fabricated. Maxillomandibular records were made by placing the occlusal rims in the oral cavity, with a freeway space of 2 mm, and using Willi's measurements (Figure 6). Using the interocclusal record, the working casts were mounted in a simple articulator to evaluate dentition and occlusal vertical dimension (OVD) (Figure 7).

**Figure 8:**
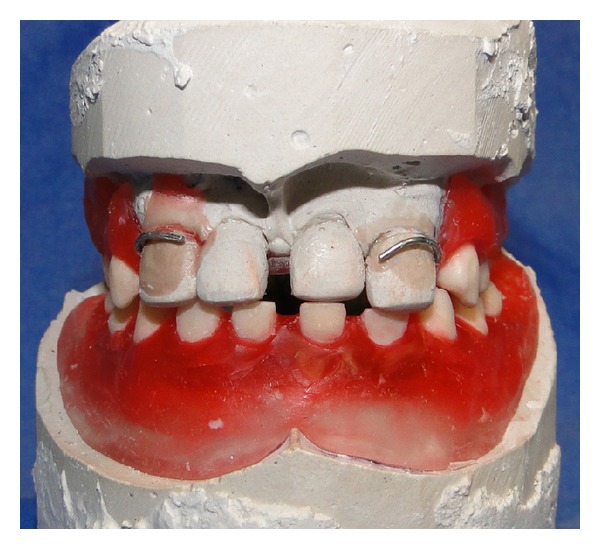
Frontal view of the casts mounted in a simple articulator with artificial teeth.

**Figure 9:**
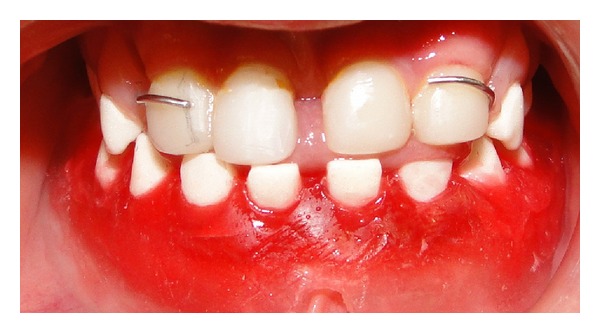
Wax try-in of the maxillary and mandibular dentures.

The artificial teeth needed for the denture were fabricated at the lab with a suitable shade of heat cure acrylic resin (Figure 8). The teeth shaped as primary teeth were arranged on the wax occlusal rim with spacing to simulate the natural spacing expected for the patients' age. The artificial teeth were arranged in wax for trial evaluation. Tooth positions, occlusal relationships, and the necessary corrections were made before processing the dentures after the prosthesis wax try-in (Figure 9).

**Figure 10:**
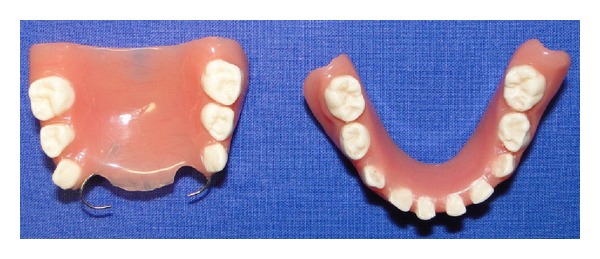
Artificial teeth in acrylic resin.

**Figure 11:**
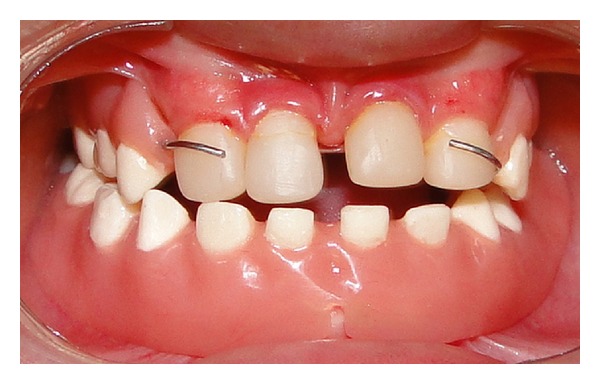
Maxillary and mandibular complete dentures in occlusion.

Later the dentures were processed with heat cure acrylic resin. The flashing errors were corrected after processing. On insertion of the denture, occlusal interferences were eliminated, and finishing and polishing of the final denture were done (Figures 10 and 11). 

After the final insertion, the patient and parents were instructed in routine oral hygiene for dentures. At recall appointments, no pressure spots were noticed, and the static and dynamic occlusion showed no interferences. Retention was excellent, and the parents reported a significant improvement of his speech and mastication. The increased self-esteem improved the socialization skills of the boy. The patient has been followed up for 12 months in our institution, and when the denture does not fit properly, a new denture will replace this one, year-by-year, if necessary, until his development stops, and the treatment plan can be reassessed.

## 3. Discussion

Several treatment strategies have been reported for the management of patients suffering from anodontia and oligodontia. Conventional removable complete or partial dentures are usually the treatment of choice for these patients [2, 6]. Providing early prosthetic care for very young completely edentulous patients seems advisable. Early intervention with complete dentures in these patients may have a profound impact by providing masticatory efficiency, swallowing ability, good phonetics, as well as improvement in esthetics [5, 6, 8–10, 12]. Also, prosthetical rehabilitation is recommended to improve both the sagittal and vertical skeletal relationship during craniofacial growth and development [1]. Hence, the problems involved in attempting to restore function and appearance are greater than usual [5], because the children's face and jaws are constantly growing and undergoing changes in dentition [11]. 

While it is recognized that early intervention is necessary to support oral normal development, there is no consensus in the literature on the ideal age for the beginning of treatment [10, 13]. However, the necessity for a proper prosthetic treatment before school age has been reported by the majority of the authors because of functional, phonational, psychological, and esthetic needs [7, 10, 13]. Khazaie et al. [14] recommend that prosthetic treatment may be carried out as early as preschool years where young children usually adapt to their use. The majority of the authors agree that if the child is cooperative, prosthetic intervention in those as young as age 2 or 3 years can be successful [1, 11, 15]. Derbanne et al. [10], after treating more than 40 patients with anodontia and oligodontia with removable prosthesis, considered that the sooner the prosthetic rehabilitation begins, the better the results will be in terms of functions, social integration, and self-esteem of the patient. Early intervention affords the child the opportunity to develop normal forms of speech, chewing, and swallowing; normal facial support; and improved temporomandibular joint function [1, 13, 15]. The prosthodontic treatment enhances the tonus of the masticatory muscles and provides normal mastication, swallowing, and regular phonation [13]. Imirzalioglu et al. [15] described a clinical case of a child that, after 1 year of the complete dentures installed, had maxillary horizontal development as well as the condylar remodeling and posterior rotation of the mandible related to the improved vertical dimension.

The technique for denture construction is similar to that for adult patients, comprising stages of preliminary and final impressions, registration of occlusion, wax try-in, and insertion of the finished denture. However, in children some modifications of the accepted techniques are usually indicated and require special attention [2]. It is necessary to simplify the technique of fabricating complete dentures in children to promote better cooperation and ensure them a positive dental experience [11]. The fabrication of prosthesis for a child requires an extensive diagnostic process together with a detailed medical and dental history. All these factors should be considered during fabrication in order to provide a prosthesis that is not only functional but also esthetic and age appropriate [11]. Retention and stability of the prosthesis are also difficult to obtain during children's growth, particularly because of the insufficient bone support, typically amorphous tooth structure, and lack of sufficient undercut zones [11, 15]. When planning dentures in these patients, care should be taken to obtain a wide distribution of occlusal load fully extending the denture base [2].

Also, special attention must be paid to the impression technique; for complete dentures, support should not be limited to the denture base area but should also include the entire vestibular sulcus reflection for a retentive base construction with sealed border [1]. Irreversible hydrocolloid material with higher viscosity is the material suggested by the majority of the authors for primary impressions, probably because it sets faster, consequently helps prevent aspiration of the impression material, and ameliorates the patient's comfort [1, 14]. Accurate impressions with proper anatomic extensions are needed to fabricate a good custom tray, which will probably help make a superior final impression [11]. For final impressions, in the presented case report, the authors preferred silicone for border molding and as an impression material because these materials are cleaner and have the required accuracy, better working time, and a fast setting time. Some clinicians have used border molding techniques with a warm green stick compound prior to making the final impression. Due to insufficient evidence, it can be argued that this technique has limited advantages for children because of the requisite time, patient discomfort related to the procedure, and potential risk of thermal injury. Furthermore, there will be a future need for a relining or remaking of the prosthesis to accommodate jaw growth [11].

The implementation of special management and motivational techniques becomes essential to maintain a good level of cooperation from these patients [3]. Regular periodic recalls of young patients with complete dentures are usually necessary, because prosthesis adjustments or replacement will be needed as a result of continuing growth and development, until more definitive implant-assisted prostheses can be delivered [2, 5, 6, 14]. Imirzalioglu et al. [15] emphasize that the continuous jaw growth and the dentition changes of children must be closely monitored. Complete dentures require regular adjustments and should be relined, rebased, or replaced when a decreased vertical dimension of occlusion and an abnormal mandibular posture are detected due to growth [1]. Therefore, frequent (3–6-month recall intervals) follow-up examinations and denture adjustments are needed. Oral prosthesis on patients at an early age represents a long-term commitment on the part of all who are involved in the process: clinician, patient, and parents [2]. Pediatric dentistry specialists form a part of the interdisciplinary team that treats these young people, providing successful clinical outcomes and proper emotional development [14].

## 4. Conclusion

Prosthetic rehabilitation in children must be performed at the earliest age possible in order to maintain the oral functions, provide normal development, and increase self-esteem improving socialization skills.

## References

[1] Tarjan I, Gabris K, Rozsa N (2005). Early prosthetic treatment of patients with ectodermal dysplasia: a clinical report. *Journal of Prosthetic Dentistry*.

[2] Paul ST, Tandon S, Kiran M (1995). Prosthetic rehabilitation of a child with induced anodontia. *The Journal of Clinical Pediatric Dentistry*.

[3] Mussa R, Esposito SJ, Cowper TR (1999). The use of colored elastomeric “O”s as a motivational instrument for patients with anodontia: report of case. *Journal of Dentistry for Children*.

[4] Pigno MA, Blackman RB, Cronin RJ, Cavazos E (1996). Prosthodontic management of ectodermal dysplasia: a review of the literature. *Journal of Prosthetic Dentistry*.

[5] Vieira KA, Teixeira MS, Guirado CG, Gavião MBD (2007). Prosthodontic treatment of hypohidrotic ectodermal dysplasia with complete anodontia: case report. *Quintessence International*.

[6] Pettit S, Campbell PR (2010). Ectrodactyly-ectodermal dysplasia-clefting syndrome: the oral hygiene management of a patient with EEC. *Special Care in Dentistry*.

[7] Paschos E, Huth KC, Hickel R (2002). Clinical management of hypohidrotic ectodermal dysplasia with anodontia: case report. *The Journal of Clinical Pediatric Dentistry*.

[8] Al-Ibrahim HA, Al-Hadlaq SM, Abduljabbar TS, Al-Hamdan KS, Abdin HA (2012). Surgical and implant-supported fixed prosthetic treatment of a patient with ectodermal dysplasia: a case report. *Special Care in Dentistry*.

[9] Kohli R, Levy S, Kummet CM, Dawson DV, Stanford CM (2011). Comparison of perceptions of oral health-related quality of life in adolescents affected with ectodermal dysplasias relative to caregivers. *Special Care in Dentistry*.

[10] Derbanne MA, Sitbon MC, Landru MM, Naveau A (2010). Case report: early prosthetic treatment in children with ectodermal dysplasia. *European Archives of Paediatric Dentistry*.

[11] Bidra AS, Martin JW, Feldman E (2010). Complete denture prosthodontics in children with ectodermal dysplasia: review of principles and techniques. *Compendium of Continuing Education in Dentistry*.

[12] Sholapurkar AA, Setty S, Pai KM (2011). Total anodontia in patient with hypohidrotic ectodermal dysplasia. Report of rare case of Christ-Siemens Touraine syndrome. *The New York State Dental Journal*.

[13] Açikgöz A, Kademoglu O, Elekdag-Türk S, Karagöz F (2007). Hypohidrotic ectodermal dysplasia with true anodontia of the primary dentition. *Quintessence International*.

[14] Khazaie R, Berroeta EM, Borrero C, Torbati A, Chee W (2010). Five-year follow-up treatment of an ectodermal dysplasia patient with maxillary anterior composites and mandibular denture: a clinical report. *Journal of Prosthodontics*.

[15] Imirzalioglu P, Uckan S, Haydar SG (2002). Surgical and prosthodontic treatment alternatives for children and adolescents with ectodermal dysplasia: a clinical report. *Journal of Prosthetic Dentistry*.

